# Neurological, Psychiatric, and Biochemical Aspects of Thiamine Deficiency in Children and Adults

**DOI:** 10.3389/fpsyt.2019.00207

**Published:** 2019-04-04

**Authors:** Shibani Dhir, Maya Tarasenko, Eleonora Napoli, Cecilia Giulivi

**Affiliations:** ^1^Department of Molecular Biosciences, School of Veterinary Medicine, University of California, Davis, Davis, CA, United States; ^2^Medical Investigations of Neurodevelopmental Disorders (MIND) Institute, University of California, Davis, Davis, CA, United States

**Keywords:** autism spectrum disorders, brain, depressive disorders, encephalomyopathies, Krebs cycle, pentose phosphate pathway, thiamine transporter

## Abstract

Thiamine (vitamin B1) is an essential nutrient that serves as a cofactor for a number of enzymes, mostly with mitochondrial localization. Some thiamine-dependent enzymes are involved in energy metabolism and biosynthesis of nucleic acids whereas others are part of the antioxidant machinery. The brain is highly vulnerable to thiamine deficiency due to its heavy reliance on mitochondrial ATP production. This is more evident during rapid growth (i.e., perinatal periods and children) in which thiamine deficiency is commonly associated with either malnutrition or genetic defects. Thiamine deficiency contributes to a number of conditions spanning from mild neurological and psychiatric symptoms (confusion, reduced memory, and sleep disturbances) to severe encephalopathy, ataxia, congestive heart failure, muscle atrophy, and even death. This review discusses the current knowledge on thiamine deficiency and associated morbidity of neurological and psychiatric disorders, with special emphasis on the pediatric population, as well as the putative beneficial effect of thiamine supplementation in autism spectrum disorder (ASD) and other neurological conditions.

## Introduction

**Figure 1:**
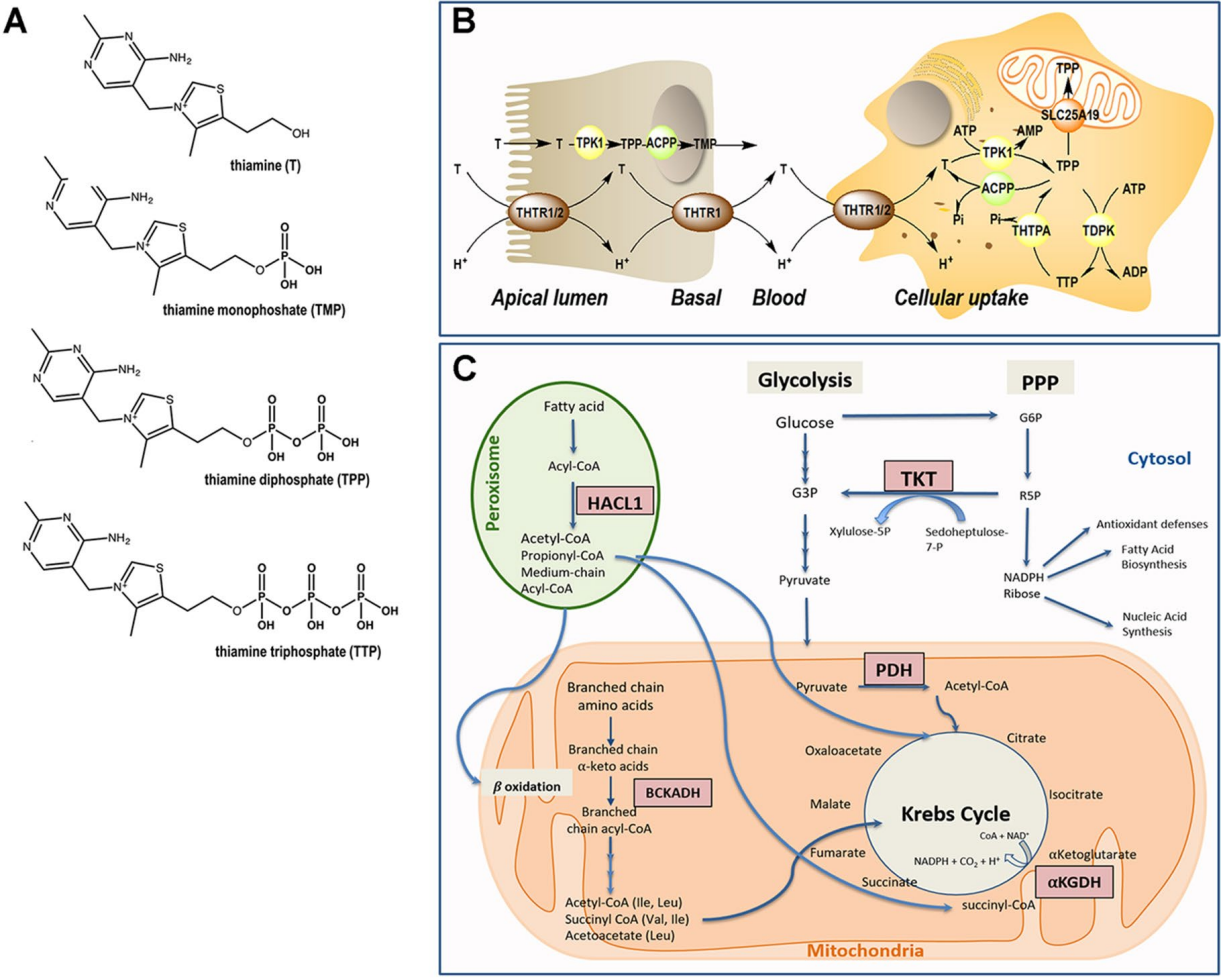
Thiamine metabolism. **(A)** Chemical structure of thiamine and phosphorylated derivatives. **(B)** Absorption and cellular uptake of thiamine. Dietary thiamine is mainly in the form of phosphate derivatives and, before absorption, these are converted to free thiamine by intestinal phosphatases. Mammalians utilize THTR1 and THTR2 to transport thiamine. The THTR1 appears to function in the micromolar range, whereas the THTR-2 appears to function in the nanomolar range. Both transporters are expressed in the human small and large intestine, and the expression of THTR1 is higher than that of THTR2, with THTR1 found at both the apical and basolateral membranes of enterocytes (somewhat higher expression at the latter compared to the former domain). Expression of the THTR2 protein was found to be restricted to the apical membrane domain only [see (5) and references therein]. Free thiamine is converted to TPP by the action of TPK1 in the cytosol and dephosphorylation of TPP to TMP and thiamine by ACPP. At higher concentrations of thiamine, simple passive diffusion takes place [see (6) and references therein]. From blood, thiamine is taken up by either transporter (THTR1 or THTR2). Both carriers are widely distributed in the body, but the levels differ in different tissues. Once inside the target cells, thiamine is phosphorylated to TPP by TPK1 and to TTP by TDPK. Both TPP and TTP can be dephosphorylated by ACPP and THTPA, respectively. TPP in the cytosol is utilized as a cofactor for TKT, but it is also required as a cofactor in the mitochondrial compartment; hence, the mitochondrial SLC25A19 exists at the inner mitochondrial membrane. TPP must be bound to HACL1 and HACL2 for its transport to the peroxisomes. Abbreviations: ACPP, prostatic acid phosphatase; ADP, adenosine diphosphate; AMP, adenosine monophosphate; ATP, adenosine triphosphate; T, thiamine; TDPK, thiamine diphosphokinase; THTPA, thiamine triphosphatase; THTR, thiamine transporter; TMP, thiamine monophosphate; TPP, thiamine pyrophosphate. **(C)** Pathways involving TPP-dependent enzymes. In the cytosol, TPP is the cofactor of transketolase (TKT), an enzyme involved in the pentose phosphate pathway (PPP). The PPP produces nicotinamide adenine dinucleotide phosphate (NADPH) and ribose 5-phosphate (R5P), essential for antioxidant defense and fatty acid synthesis, and precursor in the biosynthesis of nucleotides, respectively. In mitochondria, TPP acts as a cofactor for branched-chain α-ketoacid dehydrogenase (BCKDH) complex, pyruvate dehydrogenase (PDH) complex, and α-ketoglutarate dehydrogenase. In peroxisomes, TPP is a cofactor for 2-hydroxyacyl-CoA lyase 1 (HACL1) as part of the fatty acid α-oxidation pathway.

The essential nutrient thiamine (vitamin B1) is a water-soluble, sulfur-containing vitamin belonging to the vitamin B complex family (**Figure 1A**). Not being endogenously synthesized, the only available source of thiamine is dietary (beef, poultry, cereals, nuts, and beans). The body does not store thiamine in levels >30 mg, and the half-life for thiamine is only 9–18 days. For an average of ∼2,000 kcal consumed daily, the minimum thiamine requirement is calculated at 0.66 mg (1), although the recommended daily intake for adult men and women is 1.2 and 1.1 mg, respectively (1, 2). During pregnancy or breast-feeding, this requirement increases to 1.4 mg/day (1). In children, the recommended dietary allowance (RDA) is age-dependent and spanning from 0.2 mg (from birth up to 6 months old) to 0.6 mg (from 6 months old to 8 years old) (3). In the human body, thiamine-rich tissues are skeletal muscles, heart, liver, kidney, and brain (4).

Despite the availability of dietary thiamine in wealthy countries, thiamine deficiency represents an important and usually overlooked issue. In developed countries, the predominant use of industrial food processing often depletes thiamine content along with other vitamins and nutrients. An increased consumption of processed food in the form of simple carbohydrates, not supplemented with adequate levels of thiamine, has been named “high calorie malnutrition” (7, 8). Thus, despite the caloric density, the diet is often of poor nutrition quality and does not meet recommended dietary guidelines for micronutrient intake, making this an at-risk population for micronutrient malnutrition (8). For instance, at least 29% of obese subjects that will undergo bariatric surgery have been reported as thiamine deficient (9). This condition highlights the fine balance between adequate caloric intake and balanced nutritional diet. As thiamine is a key factor in the metabolism of glucose, an increased carbohydrate intake will proportionally increase thiamine’s dietary demand (a minimum of 0.33 mg per 1,000 kcal) (1). Thus, rather than focusing on thiamine’s RDA, it is critical to match its intake with carbohydrate consumption as well as total caloric intake.

In developing countries, thiamine deficiency remains a widespread concern due to high rates of white rice consumption (3). As home-pounding techniques are replaced with industrial rice milling and processing, essential nutrients (such as thiamine) within the bran are stripped away (10). Asian countries consume about 90% of the rice produced worldwide, fulfilling an estimated 60% of the population’s daily dietary energy intake requirement, and consequently, thiamine deficiency has become prevalent in the 15% of the adolescent population (using the most conservative approximation) (11). Thiamine deficiency may develop by ingesting diets either contaminated with thiamine-metabolizing enzymes (e.g., thiaminase) (12) or that underwent thiamine inactivation by heat and/or sulfur dioxide (13). Heavy consumption of tannin-containing or food rich in caffeine, theobromine, and theophylline (such as those present in coffee, chocolate, and tea, respectively) can inactivate thiamine, thereby compromising the thiamine status (7, 14, 15).

Other risk factors that increase the likelihood of insufficient thiamine intake include aging, economic status, eating disorders, medical conditions affecting the gastrointestinal tract, subjects receiving parental nutrition, bariatric surgery, diabetes, and alcohol abuse (9, 16–23). Unmet needs for increasing the nutritional intake of thiamine are reported during lactation, pregnancy, and increased physical activity (11, 24). During lactation, infants have increased risks of developing beriberi from newly deficient but asymptomatic mothers (11). For instance, 27% of women of childbearing age are considered thiamine deficient in Cambodia, with 38% of infants being diagnosed as severely deficient in thiamine, a critical issue that contributes significantly to the mortality of 3-month-old babies (11).

However, even in the presence of an adequate thiamine intake, its deficiency can result from genetic factors, i.e., pathogenic gene mutations in key regulators of the thiamine pathway, including thiamine pyrophosphokinase 1 (TPK1), thiamine diphosphate kinase (TDPK), thiamine triphosphatase (THTPA), and thiamine transporters (SLC25A19, SLC19A2/THTR1, and SLC19A3/THTR2; **Figure 1B**). More recently, the organic cation transporter 1 (OCT1) has been claimed to act as a hepatic thiamine transporter (25).

Regardless of the underlying cause, thiamine deficits may have severe detrimental effects, with most of the symptoms manifesting at the neurological level (24, 26). Thiamine deficiency might cause brain tissue injury by inhibiting brain energy utilization given the critical role of thiamine-dependent enzymes associated within glucose utilization (27). This is supported by the significant rate of thiamine uptake by the blood–brain barrier emphasizing the high brain demand for thiamine and the need for its supply to sustain adequate brain functions (28, 29), especially in those brain areas with both high metabolic demands and high thiamine turnover (30–32).

This review will discuss the physiological and biochemical bases of thiamine metabolism and explore the pathophysiological implications of thiamine deficiency. In particular, we will highlight the effect of thiamine supplementation as a therapeutic strategy in the management of neurological disorders, with special emphasis on the pediatric population.

## Biochemical Basis of Thiamine’s Cellular Role

### Dietary Availability, Absorption, and Cellular Uptake

As with most hydrophilic micronutrients, thiamine absorption occurs mainly in the jejunum (33). Throughout the digestive tract, dietary proteins get hydrolyzed, releasing thiamine. In the intestinal lumen, alkaline phosphatases catalyze the hydrolysis of thiamine-phosphorylated derivatives into free thiamine (34). Unphosphorylated, free thiamine at concentrations higher than 1 µM enters the enterocyte by passive diffusion, whereas at lower levels, it is transported *via* the saturable thiamine/H^+^ antiport system (thiamine transporter 1 or THTR1) through an energy-dependent process (33). Under conditions of thiamine deficiency, an upregulation of the expression of the thiamine transporter 2 (THTR2) was observed in Caco2 cells in culture, suggesting that diet can modulate the expression of this transporter (35). Within the enterocyte, thiamine is phosphorylated to thiamine pyrophosphate (TPP) by TPK1. Then, most TPP is dephosphorylated to thiamine monophosphate (TMP) to cross the basal membrane of the enterocyte. The TMP is released into the bloodstream through an ATPase-dependent transport system (36) (**Figure 1B**). Free thiamine can also reach the bloodstream *via* the thiamine transporter 2 (THTR2) located mainly at the basolateral membrane of the enterocyte. Once in blood, while very low levels of TMP and thiamine circulate free in plasma or serum, more than 90% of the phosphorylated thiamine (in the form of TPP) is present in erythrocytes and leukocytes (37). Notably, the isoform 3 of the carrier SLC44A4 has recently been described as a TPP carrier in the colon. Originally, SLC44A4 was described as a choline transporter linked to the non-neuronal synthesis of choline (38) and required to the efferent innervation of hair cells in the olivocochlear bundle for the maintenance of physiological function of outer hair cells and the protection of hair cells from acoustic injury (39). Recent evidence indicates that this carrier may mediate the absorption of microbiota-generated TPP (especially in infants) and contribute to host thiamine homeostasis (40).

The cellular uptake of thiamine from the bloodstream can be mediated by any of the two high-affinity carriers: THTR1 [encoded by *SLC19A2* (41)] and THTR2 [encoded by *SLC19A3* (42, 43)]. These transporters are ubiquitously expressed (42–44), but THTR1 is most abundant in the intestine, skeletal muscle, nervous system, and eye followed by the placenta, liver, and kidney, whereas THTR2 is located mostly in adipose tissue, breast tissue, liver, lymphocytes, spleen, gallbladder, placenta, pancreas, and brain (information collected from GeneCards). Once transported intracellularly, free thiamine is rapidly phosphorylated to TPP by thiamine pyrophosphokinase (TPK1). A second kinase, TDPK, adds a phosphate group to TPP to generate thiamine triphosphate (TTP). TPP and TTP can be dephosphorylated to, respectively, TMP and TPP by phosphatases [Prostatic Acid Phosphatase (ACPP) and THTPA, respectively; **Figure 1B**].

### Biochemical Roles of Phosphorylated Forms of Thiamine

Up to 90% of the total thiamine in the body remains in its diphosphate, metabolically active form (TPP), whereas the rest is found as TMP and TTP (45). TPP is a cofactor of several thiamine-dependent enzymes involved in carbohydrate and fatty acid metabolism, namely, cytosolic transketolase (TKT), peroxisomal 2-hydroxyacyl-CoA lyase 1, and three mitochondrial enzymes (pyruvate dehydrogenase, α-ketoglutarate dehydrogenase, and branched-chain α-ketoacid dehydrogenase complexes; **Figure 1C**). As the biochemical role of TPP is well understood, the biological significance and contribution of TTP is not entirely clear. It was previously considered to be a specific neuroactive form of thiamine, but more recently, it has been reported that TTP (which is ∼10% of the total brain thiamine pool) is involved in membrane excitability and nerve conduction by acting as a modulator of the permeability of sodium chloride channels (29, 32, 46).

#### Intracellular Location of TPP-Dependent Enzymes

##### Cytosol

In the cytosol, TPP acts as a cofactor for TKT, a key enzyme of the non-oxidative branch of the pentose phosphate pathway (PPP). This metabolic pathway generates nicotinamide adenine dinucleotide phosphate (NADPH) and ribose 5-phosphate (R5P) (47). NADPH is a key reducing agent in biosynthetic reactions and is a co-substrate of biosynthetic enzymes (fatty acid synthesis) and antioxidant enzymes such as the glutathione peroxidase–reductase system and thioredoxin peroxidases, among others. The crucial involvement of R5P in the biosynthesis of DNA and RNA highlights the critical role of thiamine in high-proliferating tissues.

Based on its role in the abovementioned biochemical pathways, it is expected that thiamine deficiency will result in increased oxidative stress and lower cell proliferation, as well as decreased fatty acid synthesis (including myelin) with severe consequences especially during brain development. Consistent with this premise, thiamine deficiency decreases TKT activity and leads to PPP impairment and reduced neurogenesis in murine cortex and hippocampus during neurodevelopment (48).

##### Peroxisomes

Peroxisomes play an important role in the catabolism of hydrogen peroxide, as well as in the shortening of very long fatty acids (which cannot undergo a direct mitochondrial β-oxidation catabolism) and α-oxidation (49). In the latter process, the TPP-dependent enzyme 2-hydroxyacyl-CoA lyase 1 (HACL1) catalyzes the cleavage of 3-methyl-branched and straight chain 2-hydroxy long-chain fatty acids (50). Phytanic acid (a 3-methyl-substituted, 20-carbon branched-chain fatty acid), unlike most fatty acids, is unable to undergo β-oxidation because of an existing methyl group in the 3-position (51). As such, it is broken down by HACL1 by an initial α-oxidation (52, 53). This branched-chain fatty acid is obtained through the diet, specifically from dairy products and red meat. The disruption of phytanic acid catabolism, due to inadequate levels of TPP, leads to triglyceride accumulation, which may cause deleterious effects such as cerebellar ataxia, peripheral polyneuropathy, vision and hearing loss, anosmia, and, in some instances, cardiac dysfunction and epiphyseal dysplasia (54). The symptoms caused by thiamine deficiency are shared by Refsum’s disease, which is caused by pathogenic mutations in *HACL1* (55). Some of the symptoms are also observed in the autosomal recessive systemic disorder Zellweger syndrome and other peroxisomal-related diseases including the neonatal adrenoleukodystrophy. Zellweger syndrome is caused by pathogenic mutations in the pexin genes, which encode for proteins essential for the assembly of functional peroxisomes. It is characterized by deficits in the peroxisomal fatty acid oxidation pathway causing severe neurological and liver dysfunction as well as craniofacial abnormalities.

##### Mitochondria

Most (∼90%) of the cytosolic TPP is transported into mitochondria *via* the mitochondrial thiamine pyrophosphate transporter [MTPPT, product of the *SLC25A19* gene (56)]. This transporter mediates the exchange of cytosolic TPP for the mitochondrial TMP; once in the cytosol, TMP is metabolized and converted back to TPP (56). In mitochondria, TPP is a critical cofactor for three enzymes, namely, pyruvate dehydrogenase, α-ketoglutarate dehydrogenase, and branched-chain α-ketoacid dehydrogenase (PDH, αKGDH, and BCKDH, respectively).

Studies in *SLC25A19* knockout mice and in carriers of the *SLC25A19* mutation, responsible for the Amish lethal microcephaly (MCPHA, see below) show, respectively, virtually undetectable and markedly reduced mitochondrial TPP levels in mouse embryonic fibroblasts and human lymphoblasts (56), which were completely restored upon addition of TPP to the PDH and KGDH complex assay mixtures (56).

###### Pyruvate dehydrogenase complex

This multisubunit complex catalyzes the TPP-dependent decarboxylation of pyruvate, generating acetyl-CoA, which then enters the Krebs cycle. The regulation of PDH activity constitutes a key metabolic switch influencing fuel choice, i.e., between fatty acid oxidation and glycolytic flux (57). The inability to adjust the choice of fuel for metabolic energy production has been proposed to underlie the “metabolic inflexibility” leading to the morbidity of metabolic disorders (58). Hence, thiamine-deficiency-mediated inhibition of the PDH complex will lock the system into the oxidation of glucose to pyruvate, leading to increases in lactate and decreases in cellular ATP production (59). As expected, in severe cases, the metabolic deficit presents as a fatal lactic acidosis in newborns, whereas in milder cases, neurological conditions may lead to structural abnormalities in the central nervous system (CNS), seizures, mental disability, and spasticity (60, 61).

###### α-Ketoglutarate dehydrogenase complex

This TPP-dependent enzyme catalyzes the formation of succinyl-CoA from α-ketoglutarate in the Krebs cycle and the consequent production of reduced nicotinamide adenine dinucleotide (NADH). Low TPP levels notably decrease αKGDH activity, thereby decreasing energy production and causing glutamate to accumulate (62), hampering oxidative metabolism and leading to neurodegeneration (63).

###### Branched-chain α-keto dehydrogenase complex

The TPP-dependent BCKDH enzyme, localized at the inner mitochondrial membrane, catalyzes one of the steps of the essential branched-chain amino acid (BCAAs; leucine, isoleucine, and valine) catabolic pathway. These amino acids are required for protein synthesis, and some of their catabolic products can be used for energy production *via* the citric acid cycle. In addition, their carbon skeletons (except Leu) can be used as precursors for gluconeogenesis (64). In a thiamine deficiency experimental model, medial thalamus was the brain region that shows the lowest levels of BCKDH with fivefold accumulation of Leu. This study suggested that the lower activity of this enzyme may be responsible for thalamic neuronal cell death observed in human cases of thiamine deficiency (65).

Similarly, a homozygous mutation in the BCKDH E1 α subunit results in the so-called maple syrup urine disease (MSUD, due to the distinct maple syrup smell to the urine as a result of keto acid buildup from unprocessed BCAA), characterized by physical and mental disabilities. A mild form of MSUD, called thiamine-responsive MSUD, is currently the only recognized variant that can be successfully treated with thiamine supplementation (OMIM 248600).

## Hierarchical Order of Thiamine-Dependent Enzymatic Deficiencies: Effect of Species, Tissue, and Subcellular Compartment

While it could be assumed that a deficiency in a given micronutrient affects its dependent enzymes equally, experimental evidence indicates otherwise. For instance, copper deficiency severely reduces cytochrome *c* oxidase activity affecting copper/zinc-superoxide dismutase to a lesser extent (66). Moreover, this hierarchical order is also tissue-specific as different brain areas (66). Similarly, in the case of thiamine deficiency, the most affected brain regions seem to be cerebellum, mammillary bodies, thalamus, hypothalamus, and brainstem in adults (1, 67). In children, MRI studies showed primary involvement of mammillary bodies as well as basal ganglia (68, 69) [preferentially the striatum and, to a lesser extent, the globus pallidus (68)], in addition to frontal lobes (69).

With regard to thiamine deficiency, Zhao et al. showed that, in mice, thiamine deprivation for 14 days led to different degrees of enzymatic deficiencies when testing for TKT, PDH, and αKGDH activities in the cortex and hippocampus (48). TKT activity was significantly more reduced in both brain regions compared to both mitochondrial enzymes PDH and αKGDH (48). Two studies reporting on a genetic mutation in SLC19A3.1 resulted in thiamine-deficient Alaskan huskies (26, 70). A greater reduction in TKT versus PDH activity was observed in the cortex. In the thalamus, however, PDH activity was affected twice as much as TKT activity (26). These studies support the concept of tissue-specific deficits of thiamine deficiency and also point to the species-specific differences that should be taken into account when making extrapolations to human studies.

In cases of severe thiamine deficiency, however, a broad enzymatic deficiency encompassing all three mitochondrial TPP-dependent enzymes would be expected to result in symptoms similar to those of the genetic disorder dihydrolipoamide dehydrogenase (DLDD) deficiency (OMIM 246900). This pathogenic mutation affects the E3 subunit, common to all three TPP-dependent dehydrogenases. Patients clinically present with lactic acidosis and neurodegeneration from inefficient oxidative metabolism, as well as increased BCAA concentrations and α-keto acids in the urine, with many of the same symptoms as MSUD, but with a more severe phenotype (71).

While BCKDH and HACL1 are (as indicated above) TPP-dependent enzymes, their activity is rarely reported in the context of thiamine deficiency, with the majority of studies focusing on TKT and PDH or αKGDH as indicators of oxidative stress (TKT) and mitochondrial dysfunction (PDH or αKGDH). However, a study by Navarro et al. noted significantly reduced BCKDH activity in the medial thalamus (compared to frontal cortex) accompanied by a fivefold increase in Leu levels following administration of a thiamine antagonist (65).

The consequences of thiamine deficiency on HACL1, however, remain mostly obscure. Recent work in HACL1-deficient mice revealed the presence of an additional TPP-dependent enzyme with lyase activity, localized in the endoplasmic reticulum, able to cleave 2-hydroxyphytanoyl-CoA (72). This process is not exactly like the one catalyzed by the peroxisomal HACL1 and involves a hydroxylation at position C2 carried out by the fatty acid 2-hydroxylase (FA2H), followed by the cleavage catalyzed by HACL1 (73). More recently, an orphan enzyme (HACL2) located in the endoplasmic reticulum known as bacterial acetolactate synthase-like was reported to cleave straight chain 2-hydroxyacyl-CoAs (74), and it is likely to be identical to the HACL1-like enzyme. A strong inverse association of C17:0 with diabetes and cardiovascular risk has been observed, suggesting a metabolic link between α-oxidation and these diseases (75, 76). More research is needed to elucidate the role of HADCL1 and HADCL2 in the context of thiamine deficiency.

## Defects in Genes Associated With Thiamine Metabolism

Pathogenic mutations in genes encoding for enzymes and transporters involved in thiamine metabolism result in symptoms similar to those found in nutrition-based thiamine deficiency and overlaps with disorders of mitochondrial dysfunction (**Table 1**). Those mutations affecting genes responsible for thiamine transporters 1 (*SLC19A2*; OMIM 249270) and 2 (*SLC19A3*; OMIM 607483) constitute the main cause of suboptimal intestinal thiamine absorption and, as a result, insufficient cellular distribution of thiamine through the body.

**Table 1:** Genetic mutations affecting thiamine metabolism.

Name of disease	Mutation	Protein	Age at onset	Clinical symptoms	Management (dose) (reference)
TRMA/Rogers syndrome	SLC19A2	THTR1	Birth to adolescence	Megaloblastic anemia, diabetes mellitus, sensorineural deafness, optic atrophy, congenital heart defects, short stature	Thiamine (50–100 mg/day) (77)
Biotin–thiamine-responsive basal ganglia disease	SLC19A3	THTR2	Birth to adolescence	Episodic encephalopathy associated with febrile illness, seizures, external ophthalmoplegia, dysphagia, gait ataxia, bilateral lesions of the basal ganglia	– Biotin (5–10 mg/kg/day), thiamine (300–900 mg) (78) – Biotin (5–10 mg/kg/day), thiamine (100–200 mg) (79)
Amish lethal microcephaly/THMD3	SLC25A19	Mitochondrial TPP carrier	Birth	Episodic encephalopathy associated with lactic acidosis and alpha-ketoglutaric aciduria, microcephaly, delayed psychomotor development, seizures, increased urinary lactate	– Phenobarbital (for seizures) and physical therapy (80) – High-fat diet (81)
Thiamine metabolism dysfunction syndrome 4/THMD4 (progressive polyneuropathy type)	SLC25A19	MTPC	Adolescence	Episodic encephalopathy associated with febrile illness, transient neurologic dysfunction, residual weakness, progressive axonal polyneuropathy, bilateral striatal degeneration	High-dose thiamine administered at the time of the study. Outcome not available (82)
Thiamine metabolism dysfunction syndrome 5 (episodic encephalopathy type) THMD5	TPK1	Thiamine phosphokinase 1	Early childhood	Episodic encephalopathy (Leigh-like) associated with high serum and CSF lactate with progressive neurologic and motor dysfunctions (gait disturbances, ataxia, dystonia, and spasticity, which, in some cases, may result in loss of ability to walk) triggered by infections. Cognitive function usually preserved; some developmental delay. Some patients may recover from some neurologic deficits; in others, the outcome is fatal.	Oral thiamine (100–200; 500 mg/day) (83, 84)

Carriers of homozygous or compound heterozygous loss-of-function or missense mutations in *SLC19A2* develop a rare condition known as thiamine-responsive megaloblastic anemia (TRMA) or Rogers syndrome (OMIM 249270). Most cases are currently found in isolated populations with consanguineous partners (85). This early-onset, autosomal recessive disorder is characterized by megaloblastic anemia, macrocytosis, diabetes mellitus, and sensorineural deafness. Other, more variable features include optic atrophy, congenital heart defects, short stature, and significant neurological deficits such as stroke and epilepsy during infancy (77, 86). The term “thiamine-responsive” refers to the observation of clinical, symptomatic improvement after administration of high doses of thiamine (86, 87).

The biotin–thiamine-responsive basal ganglia disease (BTBGD; OMIM: 607483) is a neurometabolic autosomal recessive disorder caused by a mutation in the *SLC19A3* gene, characterized by subacute encephalopathy with confusion, convulsions, muscle rigidity, ataxia, dysarthria, and dystonia, which can be fatal if left untreated. However, the disorder is completely reversible within by early treatment with biotin (5–10 mg/kg/day) in combination with thiamine [from 100–200 mg (79) to 300–900 mg (78); **Table 1**], underscoring the critical nature of a timely diagnosis. Signs and symptoms usually appear during childhood (between the ages of 3 and 10 years), although onset of the disease can vary between newborn period and adulthood (78, 88).

The Amish lethal microcephaly (MCPHA; OMIM 607196), also known as thiamine metabolism dysfunction syndrome-3 (THMD3; **Table 1**) (56), is caused by homozygous mutation in the *SLC25A19* gene. Amish type microcephaly is a severe autosomal recessive metabolic disorder characterized by severe microcephaly apparent at birth, profoundly delayed psychomotor development, brain malformations, and episodic encephalopathy associated with lactic acidosis and α-ketoglutaric aciduria (89). The clinical management is achieved by administering a high-fat diet (81), which sustains the production of ATP by mitochondria primarily through fatty acid β-oxidation, bypassing PDH to directly enter the Krebs cycle. Importantly, the metabolic similarity of MCPHA to PDH deficiency (OMIM 312070) indicates the relevance of administering a low-carbohydrate diet to patients with MCPHA to avoid a risk of exacerbating the lactic acidemia.

A less severe variant of the disease, thiamine metabolism dysfunction syndrome 4 (THMD4; OMIM 613710), is similarly caused by a homozygous mutation in the *SLC25A19* gene (**Table 1**). In THMD4, mitochondrial TPP uptake is reduced but not completely abolished. The disorder is characterized by childhood onset of episodic encephalopathy, causing transient neurologic dysfunction and a slight increase in cerebrospinal fluid (CSF) lactate levels during the acute phase of the disease. Most patients fully recover without therapeutic intervention; however, in some instances, mild residual weakness may persist (82).

Genetic defects in TPK1 (also known as THMD5) prevent the phosphorylation of thiamine to TPP, the active cofactor. It usually presents during childhood with acute encephalopathic episodes associated with increased serum and CSF lactate. These episodes result in progressive motor dysfunction (e.g., gait defects, ataxia, dystonia, and spasticity) associated with striatal, basal ganglial, and cerebellar regions of the brain, but cognition appeared to remain intact. Given that the phenotype is highly variable, it is likely that residual TPK1 activity and/or other cellular kinases (albeit at a lower extent and favored by the mass action of high thiamine doses) may also phosphorylate thiamine, thereby partly circumventing the metabolic block. This is based on the fact that i) some affected subjects improved with thiamine treatment [100–200 mg/day or 200 mg/twice daily (90, 91)] or with supplementation with thiamine and niacin, biotin, α-lipoic acid, and ketogenic diet (90), whereas others did not (83); and ii) deficiencies in other TPP-dependent enzymes, TKT and BCKADH, have not been observed in subjects with pathogenic mutations in TPK1 (83).

## Pathology of Thiamine Deficiency

As indicated above, the pathology of thiamine deficiency entails impaired energy-production from mitochondria in the form of ATP when using pyruvate-generating substrates (e.g., glucose) as well as increased oxidative stress (27). Under these conditions, glucose *via* glycolysis generates pyruvate, which cannot enter the Krebs cycle as acetyl-CoA due to the low activity of PDH. As such, pyruvate is transaminated to Ala or reduced to lactate *via* lactate dehydrogenase. This is consistent with the elevated lactate and organic acids observed in CSF, urine, and blood during thiamine deficiency (92–94).

Thiamine deficiency has been show to stabilize HIF-1α under normoxic conditions, thereby upregulating the expression of the glucose transporter GLUT1, VEGF, aldolase A, LDH, and THTR2 (95). This stabilization might be achieved by the inhibition of prolyl hydroxylase by increased oxidative stress conditions originated from the lower TKT activity within the PPP. These changes may be accompanied by increased glycolysis and fatty acid oxidation to sustain the cellular energy needs. The latter process may ensue with the ultimate formation of acetyl-CoA, skipping the PDH-catalyzed step. The increased flux of acetyl-CoA will then build up the levels of Krebs’ intermediates including fumarate and succinate (also inhibitors of prolyl hydroxylase), especially if the biotin-dependent pyruvate carboxylase cannot sustain the production of oxaloacetate, the most limiting substrate of the cycle. This also provides a biochemical explanation for the efficacy of the biotin and thiamine supplementation in the treatment of the biotin–thiamine-responsive basal ganglia disease. Although lower thiamine deficiency also affects α-KGDH activity, which is essential to maintain the levels of Glu, Asp, and GABA, its activity seems to be less affected than PDC, thus providing an operational Krebs cycle albeit at a lower efficiency. The lower activity of BCKADH can explain the higher levels of BCAA (Leu, Ile, and Val) in human fluids.

The human central nervous system has a high-energy demand, with 2% of the body mass overseeing about 20% of the total metabolic expenditure, the majority of which is spent on firing action potentials, on neuron communication, through chemical synapses, axon growth, and myelination (96). With glucose being the primary fuel for energy production in the brain, it is not surprising that mitochondrial dysfunction and the consequent impaired glucose metabolism have been associated with several neurological and neurodevelopmental conditions (97) and major psychiatric illnesses, such as depression (98) and schizophrenia (99). The neurological symptoms in thiamine deficiency are similar to defects of PDH, which most frequently present as Leigh-like syndrome with basal ganglia involvement. Therefore, the nervous system, which is highly specialized in the use of glucose for energy generation, seems to be most vulnerable to PDHC deficiency due to TPP depletion. In the brain, the lower mitochondrial ATP production will limit the maintenance of membrane potential *via* the action of the Na^+^,K^+^-ATPase, thereby compromising nerve conduction and chemical synapses. Moreover, the increased oxidative stress due to the lower TKT activity will damage critical biomolecules, initiating lipid peroxidation and oxidative damage to proteins resulting in fragmentation, posttranslational modifications, and cross-linkings. The modification of epitopes on normal, endogenous molecules may result in the activation of the microglia and immune cells, compounding oxidative stress-mediated damage. The lower TKT has additional detrimental actions: by providing a suboptimal NADPH supply, biosynthetic reactions (nucleic acids and lipids) are undermined. This will limit not only cell proliferation but also the synthesis of fatty acids critical for the myelin sheath of axons. Thus, the combination of increased lipid peroxidation and the decreased fatty acid synthesis can result in demyelination, explaining some of the neurological issues caused by thiamine deficiency.

Altered thiamine metabolism has also been linked to growth of both neuronal soma and dendritic arbors in murine hippocampal neurons in culture. The individual silencing of TPK1 or each of the TPP carriers Slc25a19 and Slc19a3 in neurons significantly decreased dendrite arborization and soma size compared to controls (100). Conversely, overexpression of TPK1, Slc25a19, and Slc19a3 significantly reversed these changes, leading to increased dendritic branch length and number as well as soma size. Notably, these pathways are independent and nonredundant, as the overexpression of any of the proteins did not improve the dendritic damage caused by suppression of the other two. These findings are indicative of the crucial role that thiamine metabolism plays in soma and dendrite growth (100), and they are consistent with the microcephaly and neuronal damage reported in cases of thiamine deficiency.

### In Adults

#### Symptoms and Consequences of Thiamine Deficiency

Thiamine deficiency can present with a broad range of neurological signs in children, such as anorexia, irritability, agitation, muscle pain, diminished or abolished deep tendon reflexes, ataxia, paralysis, and a progressively altered level of consciousness. Lactic acidosis may explain some of the generalized symptoms, including lethargy, irritability, anorexia, tachycardia, and tachypnea. These clinical manifestations are probably secondary to mitochondrial dysfunction in the heart and smooth muscle (particularly the gastrointestinal tract) and an autonomic nervous system insult (neurotransmitters). Others such as mood changes (agitation, confusion, and generalized malaise) may result from brain energy deficits as well as from a compromised synthesis of neurotransmitters (glutamate and GABA). Given the above-described varied clinical presentations, especially in children, any unexplained severe neurological signs or symptoms should raise the suspicion of thiamine deficiency [see Hiffler et al. (3) and references therein].

Generally, in blood from healthy adults, TPP levels <70 nmol/L are suggestive of thiamine deficiency [reference values: 70–180 nmol/L; https://www.mayocliniclabs.com (101, 102)]. Thiamine levels have also been assessed in CSF, in the context of Alzheimer’s disease and in instances of *SLC19A3* mutations (103, 104). Although no universally accepted reference values exist for CSF, thiamine levels of 5–7 nM are considered normal in adults (105).

Blood and CSF thiamine levels provide limited information when assessing the thiamine status of a subject, as they not necessarily reflect thiamine metabolic function (102) or a direct association to its levels in the tissues. As such, assessments of erythrocytes’ TKT and, if available, that of other tissue-specific TPP-dependent enzymes (PDH, αKGDH) are considered gold standards (24, 26). Basic TKT activity is usually expressed as units per gram of hemoglobin (g Hb), but more importantly, the percentage of activation of TKT to TPP supplementation are calculated (with 0–15% considered normal). One study reported that the HPLC detection of TPP in whole blood or red blood cells is equally effective to the TKT activation ratio at determining thiamine deficiency (37).

The TKT activation ratio (red blood cells) and/or the activities of TPP-dependent enzymes (leukocytes, skin fibroblasts, and muscle biopsies) are usually accompanied by testing the levels of serum lactate and pyruvate, BCAAs, organic acids, as well as brain imaging. The only cases in which the evaluation of free thiamine in plasma/serum and CSF seems to provide a valuable diagnostic tool is when dealing with pathogenic mutations in SLC19A3 (104). Similarly, urinary excretion of thiamine is also not a reliable method for the assessment of its bodily levels as it is dependent on its intake and absorption. Generally, it is expressed per unit of creatinine to account for renal function, and age should be taken into account as normal values differ in children [120 nmol/mmol creatinine in 1–13 years old (105)] and adults [220 nmol/mmol creatinine in >18 years old (105)].

Unfortunately, early symptoms of thiamine deficiency are not pronounced or distinctive enough to warrant a direct diagnosis. They include loss of appetite, nausea, weakness, apathy, fatigue, irritation, sleep disturbances, anorexia, and abdominal discomfort (106). Furthermore, the identification of specific clinical symptoms of thiamine deficiency is problematic because it is obscured by the contribution of other confounding conditions (comorbidities) such as infections and/or multiple nutritional deficiencies.

The clinical classification of thiamine deficiency falls usually into dry (or neuritic, characterized by polyneuropathy, reduced knee jerk and other tendon reflexes, and progressive severe weakness of muscles) and wet (or cardiac, characterized by edema of the legs, trunk, and face; high cardiac output; ventricular failure; and pulmonary congestion). This classification is based on the amount of fluid that accumulates in the body due to factors like cardiac function and kidney lesions, among others (107). However, many cases of thiamine deficiency are amply systemic and encompass the nervous and cardiovascular system, as well as the gastrointestinal tract (with symptoms spanning from anorexia, abdominal pain, and constipation) (108). Long-term consequences of severe thiamine deficiency are manifested by the appearance of hemorrhagic and/or necrotic lesions with neuronal and dendritic spine loss in the thalamus, hypothalamus, and cerebellum (109–111), as well as polyneuritis, peripheral edema, cardiac enlargement, and ophthalmoplegia (27).

When early, generalized thiamine deficiency is suspected, prompt administration of thiamine is advised and typically an effective treatment. A wide range of therapeutic approaches and thiamine doses are reported in the literature, spanning from 1.5 to 600 mg/day (112), with 10–20 mg/day as divided doses for several weeks for mild polyneuropathy and 20–30 mg/day for moderate to severe, usually until after disappearance of symptoms (106). Generally, thiamine deficiency is approached with doses of 5–30 mg/day intravenously (IV) or intramuscularly (IM), three times daily, followed by 5–30 mg/day orally until after disappearance of symptoms (112). However, this approach is notably less effective for individuals with chronic forms of thiamine deficiency-related disorders involving encephalopathies (see the section Wernicke Encephalopathy) (113) or TPK1 deficiencies. In the latter case, it would be worth to explore a treatment directly with TPP; however, it is not clear whether this phosphorylated thiamine form would cross the blood–brain barrier and/or reach subcellular targets such as PDH.

#### Wernicke Encephalopathy

Wernicke encephalopathy (WE) is a thiamine-deficiency-related acute neuropsychiatric condition, often associated with alcohol abuse (114). It is characterized by ataxia, loss of muscle coordination, memory loss, confusion, and ocular abnormalities (ophthalmoplegia). Failure to timely diagnose the disease and the lack of an appropriate therapy result in death in 17% of the cases, while 84% will undergo permanent brain damage involving severe short-term memory loss and hallucinations [or Korsakoff’s syndrome (KS)], residual syndrome in patients previously affected with WE (115). Because of the close association between these two conditions, they often get referred to as Wernicke–Korsakoff syndrome (WKS). Recently, it has been suggested that WE may be underdiagnosed (116), with similar incidence in both adults and children (117), with ratification of the diagnosis in 0.4–2.8% of *postmortem* cases, but recognized only in 32% and 6% of patients with and without alcoholism, respectively. In particular, it would be critical to consider WE in any patient showing two of the following characteristics: nutritional deficiency, cerebellar or oculomotor abnormalities, or impaired mental state or memory (116). Only 16% of the WE cases are expected to fully recover after thiamine supplementation. As oral absorption is highly variable and patient-dependent (116), parenteral treatment is highly recommended with daily doses of 100–200 mg thiamine (IV) for non-alcoholics and 500 mg (up to three administrations daily) for alcoholics (114).

A substantial body of literature has been published in the past >50 years regarding WE. A detailed description of the clinical features, pathophysiology, and treatment of this syndrome is beyond the scope of this review.

#### Depression

A number of studies have shown an inverse association between thiamine levels and symptoms of depression in adults. A cross-sectional study on >1,500 elderly Chinese (age 50–70 years) showed that subjects with lower levels of erythrocyte thiamine (likely representing TPP bound to TKT) displayed more severe symptoms of depression (118). In this report, although no apparent differences were noted between individuals with and without depressive symptoms in dietary thiamine intake, the authors could not exclude errors in dietary assessment related to differences in food processing (118). In addition, other factors like diabetes, altered hormone profiles, and increased alcohol consumption observed in some individuals with depressive symptoms could play a critical role in the bioavailability of thiamine (118).

A direct correlation between thiamine deficiency and symptoms of major depressive disorder (MDD) had been previously found in a population of 74 individuals with history of malnutrition (119). These findings were confirmed by another study involving 118 geriatric patients (120), although in this particular instance, no information about dietary thiamine intake was provided.

Additionally, Ghaleiha and colleagues explored the interventional effect of thiamine supplementation in thiamine-deficient subjects with MDD in a 12-week, randomized, double-blind clinical trial (121). The study concluded that symptoms of depression improved significantly in subjects with MDD following 6 weeks of thiamine supplementation compared to placebo.

Finally, positive mood changes (clearheadedness, increased energy and appetite, improved sleep patterns, and decreased fatigue) have been observed following thiamine supplementation in healthy elderly (16) as well as younger (122) women, compared to the same population assigned to the placebo group. Interestingly, while in the elderly population, a marginal deficiency of thiamine was reportedly due to the lack of a national thiamine enrichment policy for grains and cereals at the time of the study, in the case of younger women, a normal thiamine intake was recorded for all participants at baseline.

### In Children

In children, thiamine deficiency presents with acute symptoms and very rapid onset, and in the vast majority of cases, it affect infants who are breast-fed by thiamine-deficient mothers or with thiamine-deficient formulas.

A pivotal study that highlighted the crucial role of thiamine as an essential nutrient for infant development and discussed how TD phenotype may be confused or overlooked with other diseases was based on an epidemiological investigation on 24- to 39-month-old children presenting repeated vomiting, irritability, and lethargy, without major neurological abnormalities. All children who had consumed the same soy-based, thiamine-deficient formula (<0.5 mg/g formula) suffered from language and motor development deficits compared to sex- and age-matched children fed with other milk-based diets (123). Another study on the acute and long-term effects of thiamine-deficient formula-fed children who were not promptly diagnosed reported that a subset of patients (36%) died of cardiac and respiratory complications (124), and the remaining children developed intellectual disabilities, neurodevelopmental delay, and major motor dysfunction by the age of 9 (124). Recently, in another study by Harel et al. (125), more than 50% of a preschool children cohort, who were exposed to a thiamine-deficient diet for more than 1 month during the first 24 months of life, developed movement and motor skills difficulties compared to less than 10% among children with appropriate thiamine intake.

#### Infantile Thiamine Deficiency

Infantile thiamine deficiency (ITD) manifests after 2–3 weeks of thiamine deprivation with initially milder symptoms (refusal to eat, vomiting, and irritability), followed by rapid deterioration, with a high fatality rate (106). Newborns who are severely thiamine deprived may develop infantile beriberi. The disorder is rare in developed countries, as it can be readily treated with thiamine supplementation (126). However, it remains a concern in developing countries among infants breast-fed by thiamine-deficient mothers or fed with thiamine-deficient formula. Without proper thiamine supplementation, the deficiency is characterized by a rapid onset of symptoms involving vomiting, diarrhea, tachypnea, convulsions, ataxia, paralysis, cardiac dysfunction, and heart failure. In many cases, fatality occurs quickly (127). Those children who survive thiamine-deficiency-related WE [see the section Wernicke Encephalopathy and Vasconcelos et al. (128)] continue to live with developmental disabilities including severe epilepsy, language impairment, loss of motor function (to varying degrees), and mental retardation (mild to profound) (124).

In two separate case reports, one newborn (2 months old) with early infantile Leigh-like *SLC19A3* gene defect (129) and one (30 days old) with THTR2 deficiency (130) responded dramatically differently to thiamine supplementation. In the first case, the 2-month-old infant initially presenting with fever, feeding difficulties, and complex partial seizures (129) subsequently developed a severe encephalopathy. MRI showed abnormalities in both cerebral hemispheres as well as in brainstem and cerebellum, basal ganglia, and thalamus. A diffuse volume loss was also noted in both hemispheres. At the biochemical level, metabolic acidosis was diagnosed. Despite a prompt therapeutic regimen consisting of thiamine (37.5 mg/kg) and biotin (10 mg/kg) twice daily, the patient’s conditions never improved and he died at 4 months of age. The lack of response to treatment in this instance seems to suggest that its effectiveness is completely halted in the presence of a null mutation leading to a complete loss of function of the thiamine transporter in the early-onset form [see Table 1 in Alfadhel (129)].

In the second case report, the infant showed acute mitochondrial encephalopathy, accompanied by increased level of lactate in the blood and cerebrospinal fluid as well as abnormal levels of α-ketoglutarate in the urine. Cortico-subcortical lesions were visualized by MRI, some of which seemed to have appeared in the perinatal period (130). Within 48 h after administration of thiamine (100 mg/day), biotin (10 mg/day), and carnitine (300 mg/day), a remarkable improvement was observed in both clinical and biochemical outcomes (130). After discontinuing biotin, the patient remained stable for 6 months on thiamine supplementation (20 mg/kg/day) (130).

It is now widely established that less severe cases of ITD can be treated (to varying degrees) with early thiamine supplementation. In general, therapeutic recommended doses are 10–25 mg/daily IV or IM for 2 weeks, or 10–50 mg/daily orally, followed by 5–10 mg/daily for 1 month (112).

#### Autism Spectrum Disorders

More recently, thiamine-based treatments have gained a great deal of attention as a therapeutic strategy for other developmental and neurological disorders linked to mitochondrial dysfunction. One such disorder is autism spectrum disorder (ASD), a group of complex neurological and developmental disabilities, with a prevalence of 1 in 54 and 1 in 252, respectively, for males and females age 8 years in the United States (131). “Spectrum” refers to the wide heterogeneity and range of symptoms within three characteristic patterns: atypical cognitive profile, executive dysfunction, and unusually restricted or repetitive behavior (131). Individuals with ASD show severe cognitive and verbal impairments and experience challenges in the development of critical social communication skills.

Limited findings are available on the link between thiamine bioavailability and the development of autism. However, studies show that the consumption of herbal supplements rich in thiaminase during pregnancy (132) or alcohol exposure *in utero* (133) may predispose a fetus to thiamine deficiency during pregnancy and increased risk for ASD. A study conducted on rat pups nursed by thiamine-deficient dams displayed comparable findings, in which the offspring exhibited behavioral abnormalities consistent with ASD symptoms (134).

A number of hypotheses have been put forward on the pathophysiological mechanisms linking thiamine deficiency and ASD (135), including i) increased apoptosis, due to the link of thiamine with p53 (136, 137), Bcl-2 (138, 139), and caspases (140); ii) deregulation of the serotoninergic system (141, 142); and iii) increased oxidative stress, due to the putative role of thiamine in prostaglandin expression, decreased lipid peroxidation as well as expression of nitric oxide synthase (143, 144), and its involvement as a cofactor for TKT in the PPP, master regulator of NADPH homeostasis.

More interestingly, a subset of children with ASD has been characterized by PDH deficiency and mitochondrial dysfunction (145, 146), suggesting that thiamine deficiency may also ensue in PDH deficits or that a borderline thiamine intake would compound the preexistent PDH deficit, precipitating energy metabolism and leading to ASD in genetically predisposed individuals. Furthermore, a more recent study has shown decreased TPP levels in plasma from ASD children compared to healthy controls (147), suggesting either lower consumption or an impaired absorption from the gastrointestinal tract, providing a link between thiamine metabolism and the gut microbiome, which has been considered in the context of ASD (148). While there is no evidence to suggest that a sole deficiency in thiamine may result in ASD, it could certainly be a contributing element in concert with other epigenetic factors in the presence of an already predisposed genetic background.

Based on the finding that children with ASD seem to have an impairment in the catabolism and excretion of thiol-containing heavy metals, Lonsdale et al. proposed that thiamine supplementation may be indicated for all children with ASD (149), with the idea that heavy metal toxicity can hamper TKT activity, increase oxidative stress, and, in turn, disrupt thiamine homeostasis. Hence, thiamine supplementation may, to some extent, improve carbohydrate metabolism, mitochondrial function, and brain energy production (150). In that study, children with ASD were administered a thiamine derivative, bi-daily for 2 months and noted improved communication skills, sociability, sensory/cognitive awareness, and behavior in 8 out of the 10 children studied as well as reduction in urine arsenic and mercury levels (149). In a separate, double-blind, placebo-controlled study, researchers noted decreased oxidative stress, increased ATP in plasma and NADH and NADPH production in red blood cells, and improved PGI-R (Parent Global Impressions—Revised) scores in hyperactivity, tantrum, and receptive language categories in autistic children administered vitamin/mineral supplementation, which included thiamine, over a 3-month period (151).

These pilot studies provide some support for a thiamine-based approach as a therapeutic intervention for ASD. However, further studies are warranted to clarify the role and specificity of thiamine supplementation for the management of the diverse and extensive range of ASD features.

#### Depression in Children and Child-Bearing Women

To our knowledge, to date, only one study has investigated the contribution of thiamine levels to pediatric depression (152). Surprisingly, and opposite to findings in adults, this study found a direct correlation between thiamine intake and depressive symptoms in Spanish schoolchildren (7–9 years old).

Although a fairly common condition among teenagers, little is known about depression and its treatment in children 12 years old and younger (153). Depression in children is often overlooked and oftentimes mistaken by teachers and parents and labeled as laziness, learning disabilities, or belligerence (154). Similarly, the “acting out” behavior observed in pediatric depression is often labeled as bullying, rebellion, or even psychotic behavior (154).

The effects of thiamine supplementation might be significant as palliative treatment in postpartum depression (PPD) and have a critical role on the infant’s subsequent cognitive development. PPD has been associated with an increased risk of developing learning disabilities, Attention-Deficit/Hyperactivity Disorder (ADHD), and anxiety disorders in toddlers, which makes PPD a critical concern for both mother and infant (155). Nikseresht et al. investigated the therapeutic effects of Zn^2+^, Mg^2+^, and TPP supplementation in a PPD mouse model. Improvements in depressive symptoms and anxiety-like behaviors (tested by forced swimming test and elevated plus-maze), as well as total antioxidant capacity, were noted in the dams when the nutrients were administered on postpartum day 3 (156). Taken together, these findings indicate that thiamine supplementation may be a promising therapeutic strategy for PPD. However, future studies should address the role of thiamine in PPD independently and whether supplementation in instances of PPD positively impacts infant development.

## Conclusions and Future Perspectives

Thiamine deficiency, regardless of the underlying mechanism (genetic or epigenetic), impairs critical TPP-dependent enzymes involved in intermediary metabolism and antioxidant defenses. The consequent decrease in ATP production and increased oxidative stress result in neurological deficits, which become especially detrimental during critical windows of neurodevelopment. In the recent years, thiamine supplementation has been contemplated, with some success, as a therapeutic approach for neurodevelopmental disorders, including ASD and major psychiatric disorders (i.e., depression). However, the vast heterogeneity of the pathogenic mechanisms underlying these conditions, along with the difficulty of diagnosis and the assumption that diets from developed countries supply enough thiamine to match the caloric intake (including perinatal periods), has not prompted enough research to address the role and effects of thiamine supplementation as a therapeutic strategy in the context of neurodevelopment/neurodegeneration. Of note, and an often overlooked issue, is the lack of incorporation of antioxidants into supplemental treatments that are deemed critical to minimize the thiamine-deficiency-dependent oxidative-stress-derived damage. This will also allow to shed light on the contribution of oxidative stress versus mitochondrial dysfunction to the morbidity of neurodegenerative disorders.

## Author Contributions

SD and MT equally contributed to the writing of the manuscript. EN contributed to writing and edited the text. CG conceptualized the paper, contributed to writing, and edited the text.

## Conflict of Interest Statement

The authors declare that the research was conducted in the absence of any commercial or financial relationships that could be construed as a potential conflict of interest.
